# The anti-cancer transition-state inhibitor MTDIA inhibits human MTAP, inducing autophagy in humanized yeast

**DOI:** 10.1242/dmm.052173

**Published:** 2025-06-30

**Authors:** Namal V. Coorey, Isaac Tollestrup, Peter W. Bircham, Jeffrey P. Sheridan, Gary B. Evans, Vern L. Schramm, Paul H. Atkinson, Andrew B. Munkacsi

**Affiliations:** ^1^ School of Biological Sciences, Victoria University of Wellington, Wellington 6012, New Zealand; ^2^ Ferrier Research Institute, Victoria University of Wellington, Wellington 6012, New Zealand; ^3^ Centre for Biodiscovery, Victoria University of Wellington, Wellington 6012, New Zealand; ^4^ Department of Biochemistry, Albert Einstein College of Medicine, Bronx, New York 10461, USA

**Keywords:** Autophagy, Betweenness centrality, Chemical biology, Chemical genetics, Drug−drug synergy, Network analysis, Nucleoside/nucleotide metabolism, Synthetic lethality, Transition state analogs, Yeast genetics

## Abstract

Methylthioadenosine-DADMe immucillin-A (MTDIA) is a transition-state analog that potently inhibits the human protein 5′-methylthioadenosine phosphorylase (MTAP) at picomolar concentrations and elicits anti-tumor activity against lung, prostate, colon, cervical, head and neck, and triple-negative breast cancers in cell and animal models. The anti-cancer mechanisms of MTDIA involve elevated methylthioadenosine levels but are not fully understood. The yeast protein MEU1 is functionally equivalent to human MTAP. To gain further understanding, we performed chemical genetic analyses via gene deletion and GFP-tagged protein libraries in yeast that express a member of the human equilibrative nucleoside transporter (ENT) family to permit MTDIA uptake. Genomic and proteomic analyses identified genes and proteins critical to MTDIA bioactivity. Network analysis of these genes and proteins revealed an important link to ribosomal function, which was confirmed by observing reduced levels of ribosomal subunit proteins. Network analysis also implicated autophagy, which was confirmed by analyzing intracellular trafficking of GFP-Atg8 and Phloxine B viability. In yeast, a comparable effect occurred after deletion of MEU1, indicating a single target for MTDIA in yeast. Overall, our yeast model reveals specific components of the ribosome as well as induction of autophagy as integral mechanisms that mediate the bioactivity of MTDIA.

## Teaser

**Summary:** A new model system enables genomic and proteomic analyses to identify genes, proteins and pathways integral to the bioactivity of MTDIA, a potent anti-cancer transition-state analog and inhibitor of human MTAP.

## INTRODUCTION

Polyamine biosynthesis has been long proposed as a target for cancer therapy, as polyamine levels are elevated in proliferating cells (Gerner and Meyskens, 2004; Holbert et al., 2022; Wallace, 1996). Inhibiting the polyamine biosynthesis pathway with inhibitors of ornithine decarboxylase 1 (ODC1, also known as ODC) had limited effect because of rapid cellular turnover of the target ODC1, poor transport of the inhibitor, poor target affinity or because of compensatory mechanisms that increase polyamine transport (Casero et al., 2018). Within the polyamine biosynthesis pathway, 5′-methylthioadenosine phosphorylase (MTAP) functions as the only enzyme to catabolise the 2 mol of 5′-methylthioadenosine (MTA) formed with every mol of spermine (Della Ragione et al., 1986). MTA is a potent inhibitor of spermidine and spermine synthases, and must be hydrolyzed to prevent product inhibition (Pajula et al., 1979; Raina et al., 1984). MTAP converts MTA to adenine and 5-methylthioribose-1-phosphate, that are transformed to ATP and methionine, respectively, to yield S-adenosyl methionine (SAM), the primary methyl donor for metabolic and regulatory protein methylation reactions (Backlund and Smith, 1981; Finkelstein, 1990; Lu, 2000). Because MTAP is closely associated to polyamine biosynthesis, recycling of SAM and the regulation of inhibitory MTA levels, it has also been proposed as a target for cancer therapy (Basu et al., 2007).

**Fig. 1.**
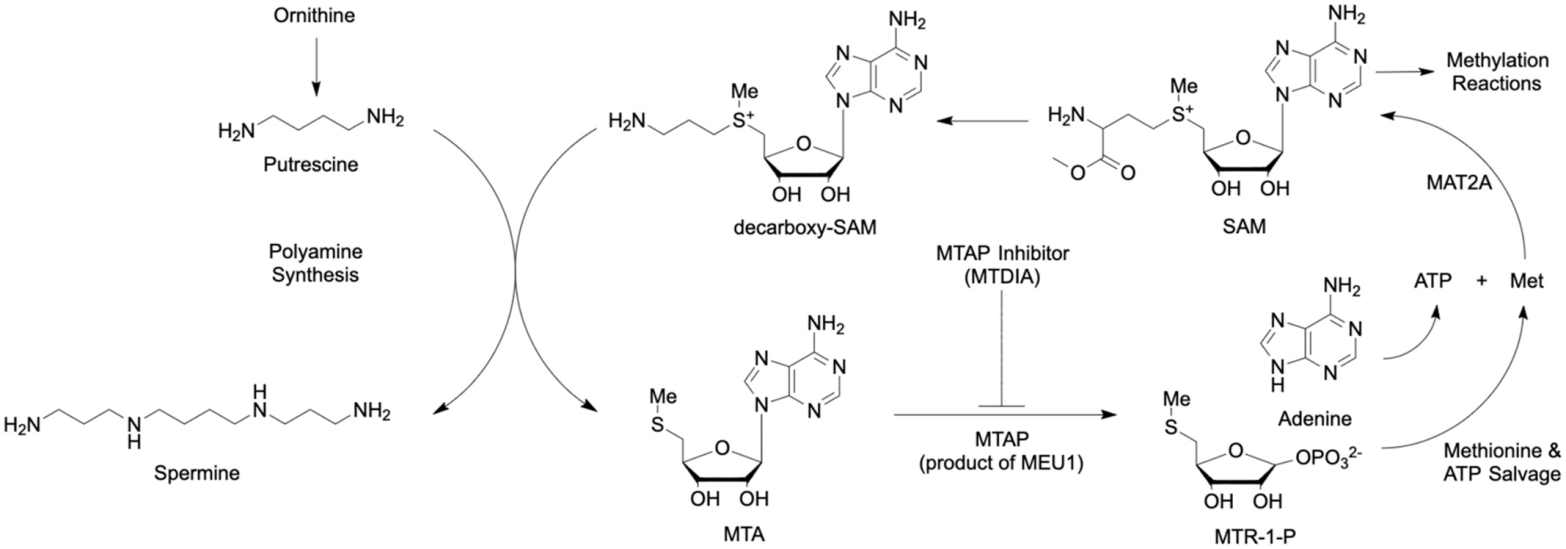
**The current working model for the MTDIA anti-cancer mechanism of action is conserved from yeast to humans.** MTDIA is an inhibitor of 5′-methylthioadenosine phosphorylase (MTAP in humans, MEU1 in yeast), resulting in 5′-methylthioadenosine (MTA) accumulation and disruptions to methionine metabolism and polyamine synthesis. MTAP catalyzes the phosphorolysis of MTA to form adenine and 5-methylthio-α-D-ribose 1-phosphate (MTR-1-P) that, ultimately, can be converted to S-adenosyl-L-methionine (SAM).

Methylthioadenosine-DADMe immucillin-A (MT-DADMe-ImmA, also known and hereafter referred to as MTDIA) is a potent picomolar inhibitor of human MTAP, acting as a mimic of the enzymatic transition-state (Fig. 1) (Schramm, 2011). MTDIA has a dissociation constant (*K*_d_) of 86 pM for human MTAP (Singh et al., 2004). Preclinical efficacy has been demonstrated in mouse xenografts of human lung, prostate, colon, cervical, head and neck, and triple-negative breast cancers, as well as *in vitro* efficacy in various human cancer cell lines (Basu et al., 2007, 2011). In animals, MTDIA mimics the loss of MTAP, leading to accumulation of MTA, inhibition of arginine protein methyltransferase-5 (PRMT5) and the potential of reducing levels of SAM, spermidine and spermine. One or more of these processes are involved in mediating apoptosis and G2/M arrest, loss of mitochondrial membrane integrity, and cleavage of poly-ADP ribose polymerase (PARP) and caspases (Basu et al., 2011); however, the genetic mechanisms underlying these processes are not fully understood.

To elucidate the genetic mechanisms of MTDIA-initiated cell death, we employed the well-established genetic model of *Saccharomyces cerevisiae* (baker's yeast). Such analyses in yeast have elucidated the mechanisms of thousands of compounds (Hillenmeyer et al., 2008; Lee et al., 2014). Since the yeast system lacks broad-substrate nucleoside transporters, we constructed gene-deletion and GFP-tagged protein libraries of yeast strains expressing the human solute carrier family 29 member 1 (SLC29A1); also known as equilibrative nucleoside transporter member 1 (ENT1), hereafter referred to as hENT1]. Genetic and chemical genetic interactions were identified by analyzing cell growth and protein abundance/localization phenotypes. Networks were then assembled and analyzed for centrality metrics to identify the key genes, proteins and pathways that mediate MTDIA bioactivity. Together, these analyses provide insight into the mechanistic complexity mediating the anti-cancer activity of MTDIA, particularly regarding involvement of the fundamental process of autophagy.

## RESULTS

### Heterologous expression of a human gene results in nucleoside uptake in yeast

**Fig. 2.**
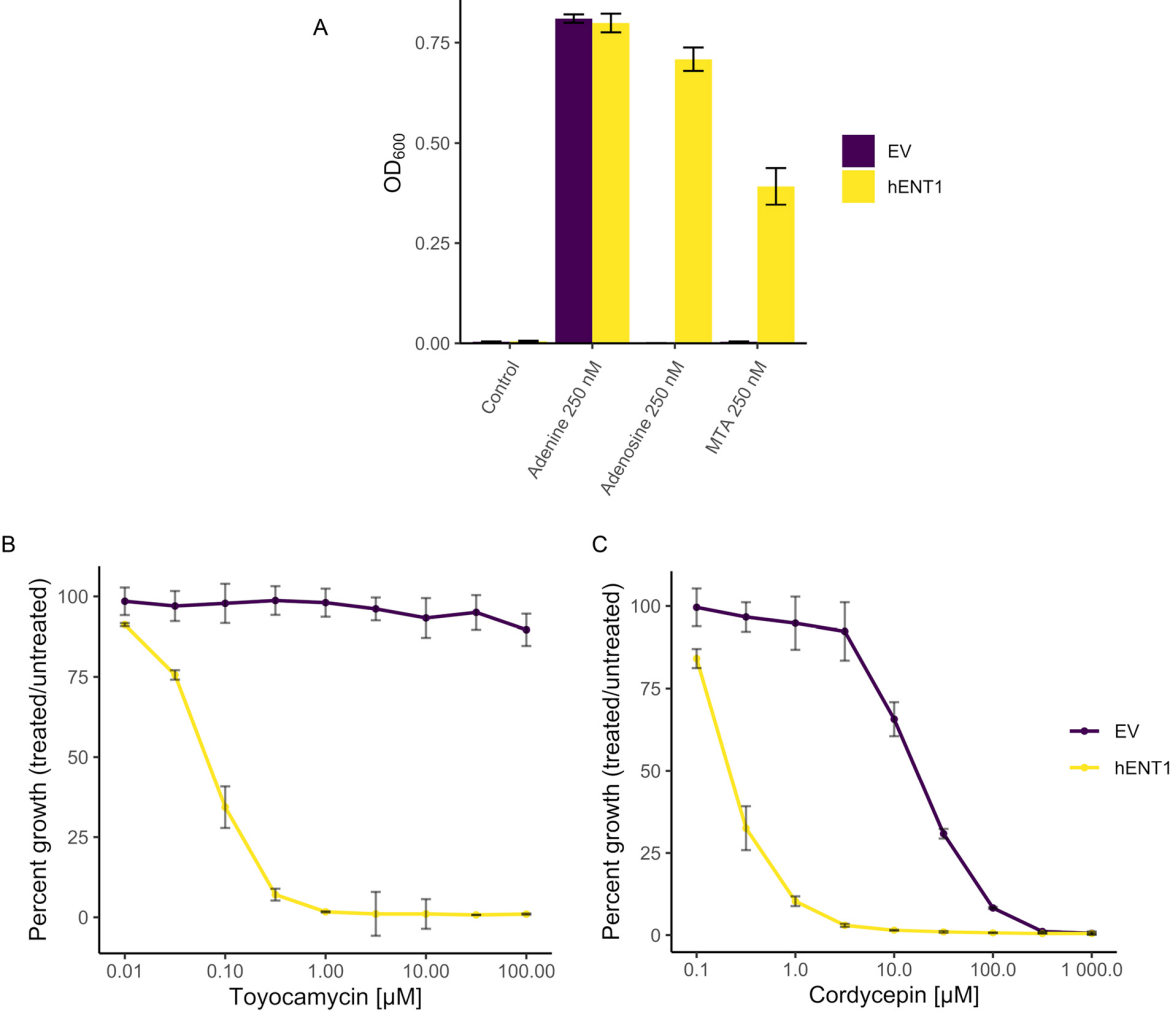
**Heterologous expression of hENT1 permits nucleoside uptake.** (A) Growth of *ade2Δade3Δ* in Y7092 (an adenine and methionine auxotroph) expressing empty vector (EV) or hENT1 was quantified in the presence of adenine, adenosine or 5′-methylthioadeonisine (MTA) after 48 h growth. (B,C) Growth of Y7092 (a methionine auxotroph) expressing EV or hENT1 was quantified in the presence of varying concentrations of toyocamycin (B) or cordycepin (C). Cells were first cultured overnight in SD medium lacking uracil but supplemented with raffinose as the carbon source (SD-U+R), then subcultured in SD medium lacking adenine and uracil but supplemented with raffinose and galactose as the carbon source (SD-AU+RG) (panel A) or in SD medium lacking uracil with raffinose and galactose as the carbon source (SD-U+RG) (panels B,C) to 5×10^5^ cells/ml, treated with nucleosides or nucleobases and incubated at 30°C. Cell growth was quantified hourly via OD_600_ readings. Plotted is the growth of treated and untreated cells (in %) at time points when untreated cells were at mid-log. Results shown in each panel represent the mean±s.d. for three biological replicates.

Yeast cells lack the orthologous plasma membrane transporters present in humans that permit the uptake of exogenous purines, pyrimidines and derivatives, including MTDIA and MTA (Young et al., 2013). The heterologous expression of galactose-inducible human equilibrative nucleoside transporter 1 (hENT1) in *S. cerevisiae* (Griffiths et al., 1997; Vickers et al., 1999) permits the uptake of pyrimidines, thymidine and uridine, and of derivatives 3′-deoxy-3′-fluorothymidine and 5-bromodeoxyuridine (Paproski et al., 2008; Vernis et al., 2003) and, in humans, also transports purine and purine derivatives (Young et al., 2013). To achieve uptake of MTDIA, hENT1 was expressed in *S. cerevisiae*, and the uptake of purine nucleosides and derivatives was assessed. While the adenine and methionine auxotroph strain (*ade2Δade3Δ* in Y7092) was inviable in adenine-free medium independent of hENT1 expression, growth of this strain occurred with hENT1 expression in adenine-free medium that had been supplemented with adenosine or 5′-methylthioadenosine (MTA) (Fig. 2A). These results demonstrate that hENT1 expression in yeast permitted the uptake of MTA, which is consistent with a previous report in that hENT1 expression in yeast permits the uptake of several nucleobases, including adenine (Yao et al., 2011).

To validate that Y7092 yeast cells expressing hENT1 take up nucleosides, we investigated their sensitivity to the toxic nucleosides toyocamycin and cordycepin. Relative to empty vector (EV), toyocamycin at concentrations between 1 and 100 µM showed no growth inhibition, whereas expression of hENT1 permitted growth inhibition due to toyocamycin at low nanomolar concentrations (IC_50_=6.6 nM) (Fig. 2B). While cordycepin was bioactive in Y7092 cells (IC_50_=17 µM), its bioactivity was improved ∼85-fold with concomitant expression of hENT1 (IC_50_=2 nM) (Fig. 2C). These results demonstrate that hENT1 allows the uptake of nucleosides in our yeast system.

### MEU1 – the yeast orthologue of MTAP - is essential when MTA is the only exogenous source of sulfur

**Fig. 3.**
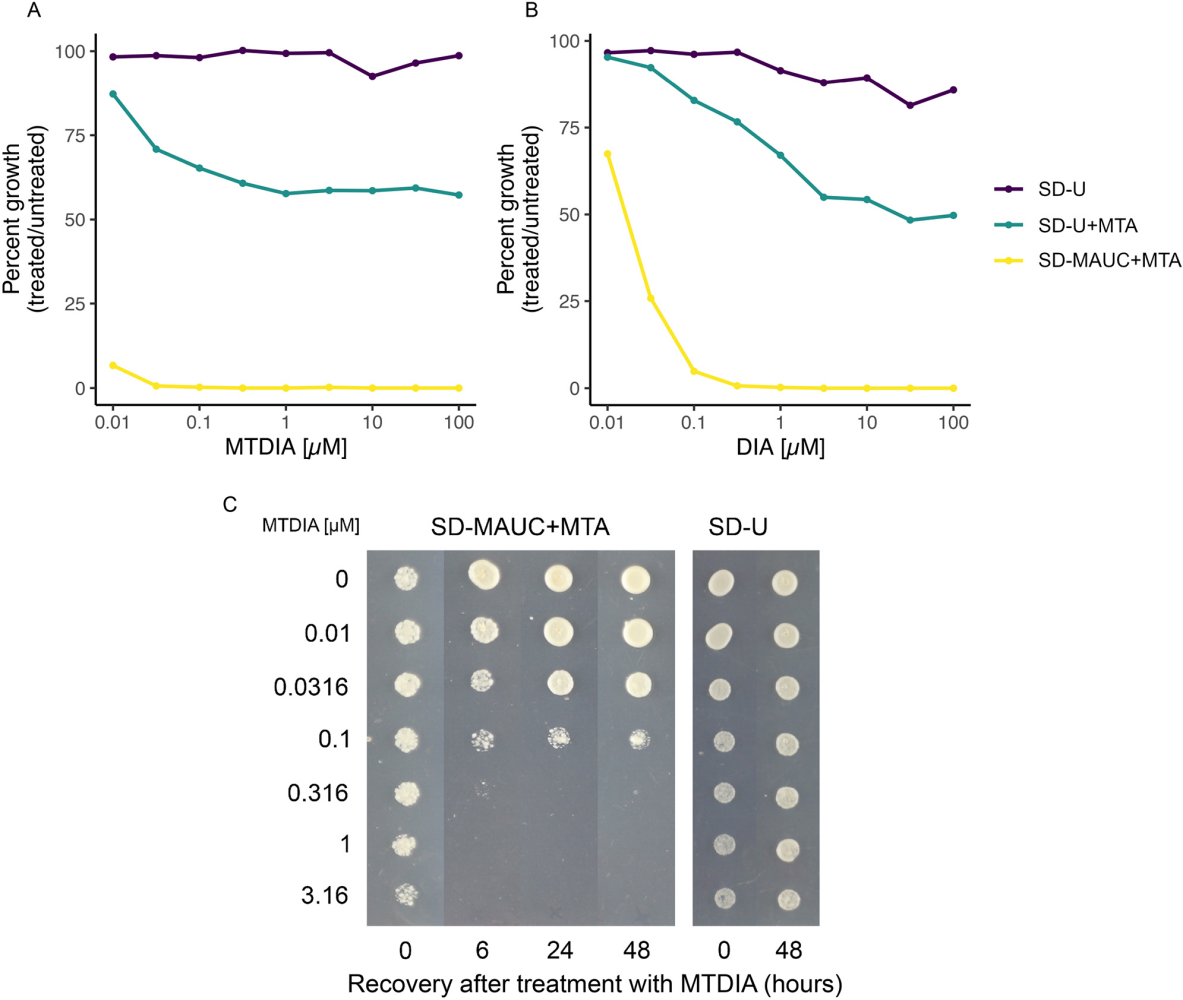
**MTDIA is a potent, cytotoxic inhibitor of yeast MTAP when methionine salvage is essential.** (A,B) Growth of Y7092 cells expressing hENT1 (Y7092+hENT1) was quantified in MTA-supplemented medium lacking uracil (SD-U) for selection of hENT1 with or without methionine, adenine and cysteine (the MEU1 essential condition SD-MAUC) in varying concentrations of MTDIA (A) or DIA (B). Cells were cultured overnight without galactose in SD-U with raffinose (SD-U+R) medium, subcultured in SD-U with raffinose and galactose (SD-U+RG) or SD-MAUC with raffinose and galactose (SD-MAUC+RG) supplemented±250 µM MTA, and treated with varying concentrations of MTDIA (A) or DIA (B). All cells were then incubated at 30°C, growth was quantified hourly via OD_600_ readings. Plotted is the mean growth (in %) of treated and untreated cells at time points when untreated cells were at mid-log. (C) Growth of MTDIA-treated Y7092+hENT1 cells on medium lacking MTDIA. Mid-log cells were treated at 30°C with varying concentrations of MTDIA for different lengths of time, plated on medium selecting for the Meu1 essential condition (left) or medium selecting only for the hENT1 plasmid (right), and incubated at 30°C for 48 h. Results shown in each panel are representative of three biological replicates.

Elucidating mechanisms of action for compounds can be accomplished by screening gene deletion libraries for hypersensitivity to the compounds, specifically, this requires perturbation of an essential target (Hillenmeyer et al., 2008; Lee et al., 2014; Parsons et al., 2006). Since MEU1, the yeast orthologue of *MTAP* in humans, encodes a non-essential protein, we sought to make MEU1 conditionally essential by screening methionine auxotrophic strains with the MTAP/Meu1 inhibitors MT-DADMe-immucillin A (MTDIA) or DADMe-immucillin A (DIA) in medium with or without the supplementation of MTA (the native substrate of Meu1) (Fig. 3A,B). Y7092 cells expressing hENT1 were not sensitive to concentrations up to 100 µM of MTDIA or DIA. In contrast, 40−50% growth inhibition occurred in hENT1-expressing cells treated with 250 µM MTA and 1 µM MTDIA or DIA. Since MTA is processed by Meu1 in the absence of methionine and cysteine to generate adenine and 5-methythioribose-1-phosphate in a methionine salvage pathway for which adenine is required for synthesis of SAM from methionine (Pirkov et al., 2008; Thomas et al., 2000), we then evaluated the impact of media lacking methionine, adenine and cysteine. Growth inhibition of hENT1-expressing Y7092 cells was dramatically exacerbated in synthetic dropout (SD) medium lacking uracil (to select for the hENT1 plasmid) as well as methionine, adenine and cysteine (SD-MAUC), where 10 nM MTDIA and 100 nM DIA elicited 93% and 99% growth inhibition, respectively. These results distinguish MTDIA as a more-potent Meu1 inhibitor than DIA. They additionally indicate that the heterologous expression of hENT1, MTA as the sole sulfur source and the absence of three components (the amino acids methionine and cysteine as well as the purine adenine) in our system are all required to render MEU1 as conditionally essential.

### MTDIA cytotoxicity depends on MEU1 and the ability to salvage methionine

Following identification of the condition for MEU1 essentiality, the nature of growth inhibition by MTDIA (i.e. cytostaticity or cytotoxicity) was assessed in an agar-based experiment. Y7092 cells expressing hENT1 were treated with MTDIA for various lengths of time (0, 6, 24 and 48 h) and growth recovery on medium lacking MTDIA was evaluated (Fig. 3C). The growth of cells on the essential medium condition (i.e. SD-MAUC+MTA) immediately following MTDIA treatment (i.e. the 0 h time point) was generally comparable to untreated cells for MTDIA concentrations of up to 1 µM. In contrast, cells treated with MTDIA for more than 6 h were unable to recover at MTDIA concentrations >0.316 µM. When cells were plated in SD medium without uracil and MTA (i.e. SD-U), they recovered and exhibited normal growth independent of duration or concentration of MTDIA treatment. These results indicated that MTDIA is a cytotoxic growth inhibitor as long as MEU1 remains essential, suggesting that growth inhibition is a consequence of the inability to salvage methionine.

### Synthetic genetic array analysis identifies genetic interactions with the Meu1 target of MTDIA

**Fig. 4.**
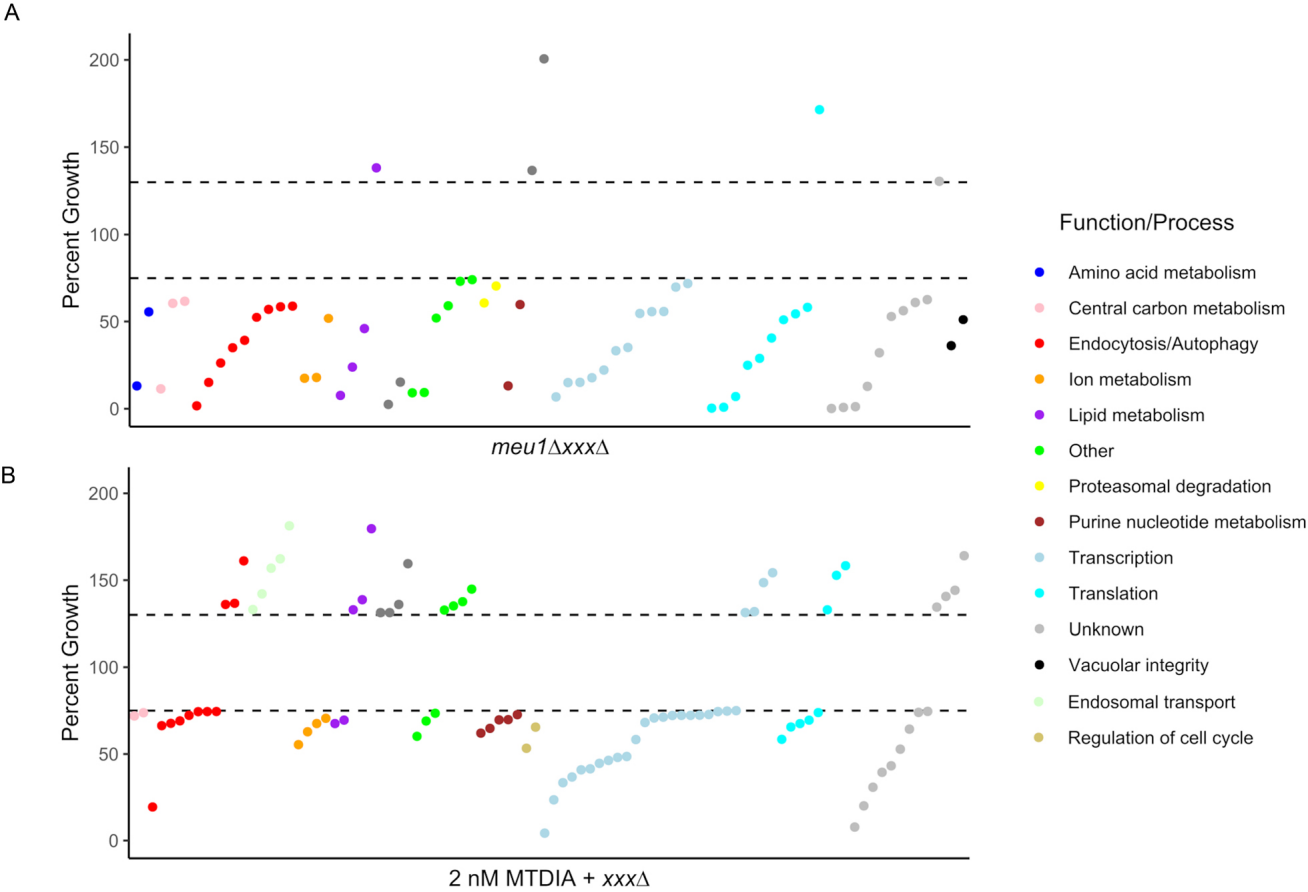
**Genome-wide analyses identify gene deletions and biological functions that are epistatic to the MTDIA target protein Meu1.** (A) Synthetic genetic array (SGA) analysis generated 4286 *meu1ΔxxxΔ* strains. Growth of these double mutant strains was compared with growth of the *meu1Δ* query strain and the *xxxΔ* strain in the deletion library. Three independent SGAs were performed by crossing the *meu1Δ* query strain with the strains of the haploid single-gene deletion library (Tong et al., 2001) and growth was quantified using Gitter (Wagih and Parts, 2014) and ScreenMill (Dittmar et al., 2010). By using liquid growth assay, genetic interactions were validated for 64 negative and six positive genetic interactions. Cells were cultured overnight, subcultured to a density of 5×10^5^ cells/ml and incubated in SD-MAUC+MTA+RG medium at 30°C. Percent growth (in %, *y*-axis) in each *meu1ΔxxxΔ* compared to the parental *meu1Δ* and *xxxΔ* was determined via OD_600_ readings when *xxxΔ* cells were at mid-log. The criterium for all strains shown was a statistically significant growth difference (either 25% growth inhibition or 30% growth improvement) of each *meu1ΔxxxΔ* compared to the parental *meu1Δ* and *xxxΔ* strains (*P*<0.05). (B) Chemical genetics analysis measured growth of 4310 *xxxΔ*+hENT1 strains on 3 nM MTDIA relative to vehicle control from biological triplicates of technical quadruplicate experiments on agar. Three independent chemical genetic screens were performed on SD-MAUC+MTA+RG agar and growth was quantified using Gitter (Wagih and Parts, 2014) and ScreenMill (Dittmar et al., 2010). Genetic interactions were validated for 61 negative and 30 positive genetic interactions in liquid growth assays. Cells were cultured overnight, subcultured to 5×10^5^ cells/ml and incubated at 30°C in SD-MAUC+MTA+RG. Growth was quantified hourly via OD_600_ readings and percent growth (in %, *y*-axis) in MTDIA compared to vehicle control was determined at time points when control cells were at mid-log. Shown A and B are the mean growth differential values for three biological replicates; mean±s.d. values are available in Tables S1 and S5. The criterium for all strains shown was a statistically significant growth difference (either 25% growth inhibition or 30% growth improvement) in each MTDIA-treated *xxxΔ* compared to the untreated *xxxΔ*, calculated using two-tailed Student's *t*-test (*P*<0.05).

Synthetic genetic array (SGA) analysis can elucidate gene−gene (genetic) interactions, providing insight into mechanisms that buffer the bioactivity of compounds (Costanzo et al., 2021). To identify the genes that are crucial in buffering MTDIA in the case of complete MTAP inhibition, MEU1 was deleted in a strain that was crossed in an SGA analysis with the haploid non-essential gene deletion library to generate 4286 haploid *meu1ΔxxxΔ* double-gene deletion mutants. Relative to the parental *meu1Δ* and *xxxΔ* single-gene deletion strains, growth of 64 *meu1ΔxxxΔ* double deletions was significantly further reduced (growth<75%, *P*<0.05) (Fig. 4A; Table S1). Additionally, six gene deletions significantly reduced sensitivity of *meu1Δ* cells (growth>130%, *P*<0.05) (Fig. 4A; Table S1). Gene ontology enrichment analyses of these 70 genes determined a significant over-representation in a multitude of biological processes (e.g. negative regulation of transcription, carbon catabolite activation of transcription, vesicle-mediated transport) (Table S2), as well as many specific functions (e.g. GTPase binding, phosphatidylinositol phosphate binding, Rab guanyl-nucleotide exchange factor activity) (Table S3). To put these functions and processes in the context of biological pathways, pathway enrichment analysis of the KEGG pathway database revealed significant enrichment in various pathways (e.g. autophagy, purine metabolism, galactose metabolism), whereby autophagy was the top-ranked pathway that was even more enriched than the proof-of-principle purine metabolism − with the metabolism of MTA being an important salvage pathway that generate purines and methionine (Table S4).

Genetic interactions of SPE3, TAT2 and AAH1 genes with *meu1Δ* were involved in amino acid/purine metabolism (Fig. 4A), which provide proof-of-principle for the expected functions of MEU1 in amino acid salvage and NAD^+^ biosynthesis. As SPE3 is required for methionine salvage from decarboxylated adenosyl methionine (Hamasaki-Katagiri et al., 1997), the double deletion mutant *meu1*Δ*spe3*Δ showed increased growth, a result consistent with compensation for polyamine deficiency when two polyamine pathway proteins, Meu1 and Spe3, are deleted. TAT2 encodes a high-affinity tryptophan transporter that is integral for synthesis of nicotinic acid mononucleotide (NaMN) in NAD^+^ synthesis and for bypassing the enzymatic step catalyzed by MEU1 (i.e. the conversion of nicotinamide ribose to nicotinamide) (Belenky et al., 2007), suggesting that a further reduction in NAD^+^ underlies the exacerbated growth defect in *meu1Δtat2Δ* cells. Since AAH1 is required for synthesis of inosine monophosphate (IMP) from adenine and deletion of MEU1 prevents the recycling of adenine from MTA, *meu1Δaah1Δ* cells must rely on adenine and inosine in the medium to synthesize IMP. It is, thus, likely that reduced IMP in this double mutant is responsible for the growth defect, which would be consistent with the poor growth of *meu1Δade2Δ* double-mutant cells compared with *ade2Δ* and *meu1Δ* single-mutant cells in the absence of adenine (Subhi et al., 2003). Together, these results validate that specific genetic interactions are integral to Meu1 inhibition by MTDIA, thus, providing insight into the molecular bases for suspected mechanisms (e.g. amino acid metabolism, transcription, translation) as well as unsuspected mechanisms (e.g. autophagy, lipid metabolism).

### Chemical genomic analysis identifies genetic interactions with MTDIA

Chemical genomic analyses have been used to identify the mechanisms mediating bioactivity of thousands of compounds, whereby the IC_20_ of compounds in wild-type cells is used to screen gene deletion mutant libraries for hypersensitivity (Hillenmeyer et al., 2008; Lee et al., 2014; Parsons et al., 2006). For assessment of MTDIA−gene interactions, growth of the *xxxΔ*+hENT1 library of 4310 unique gene-deletion strains was quantified by using medium containing 250 µM MTA and 2 nM MTDIA, a treatment that conferred a 20% growth defect in Y7092 cells, thus leaving a large window (80%) to screen a gene deletion library and identify gene deletions that interact with MTDIA. Relative to vehicle control, growth of 62 gene deletion strains was significantly reduced in response to MTDIA (growth<75%, *P*<0.05) (Fig. 4B; Table S5). These genes were involved in autophagy, carbohydrate metabolism, endocytosis, ion transport, lipid metabolism, purine metabolism, cell cycle regulation, transcription and translation. By contrast, 31 gene deletion strains were detected with significant resistance to MTDIA (growth>130%, *P*<0.05) (Fig. 4B; Table S5). These genes were involved in autophagy, endosomal transport, lipid metabolism, mitochondrial organization, transcription and translation. Gene ontology enrichment analyses of these 93 genes determined that there was significant over-representation in a multitude of processes (e.g. regulation of transcription, *de novo* inosine monophosphate biosynthesis, vesicle-mediated transport, histone deacetylation, purine nucleotide metabolism) (Table S6) as well as functions (e.g. histone deacetylase activity, DNA binding, phosphatidylinositol phosphate binding) (Table S7). Pathway analysis revealed enrichment in pathways involved in longevity, nicotinamide metabolism, fatty acid metabolism, and purine metabolism (Table S8). Notably, many of these processes, functions and pathways enriched as MTDIA interactions were also enriched with the *meu1*Δ genetic mimic, thus indicating that these results for MTDIA are an authentic consequence of MTAP inhibition.

In addition to MTDIA-sensitive and MTDIA-resistant strains, several gene deletion strains failed to grow when MTA was provided as the only sulfur source (Table S9). Several of these were noteworthy, such as MEU1, MRI1, MDE1, UTR4 and ADI1, all of which encode enzymes involved in salvage of methionine from MTA (Pirkov et al., 2008). Based on overlap with the *meu1ΔxxxΔ* results, there is additional strong evidence that MTDIA is targeting Meu1. First, the MTDIA-hypersensitive genes included gene deletion strains defective in adenine metabolism, which were involved in *de novo* biosynthesis of inosine monophosphate (IMP), while the *meu1ΔxxxΔ* strains with exacerbated growth defects were also involved in the salvage of IMP from adenine. In both cases, Meu1 inhibition with MTDIA or after deletion of MEU1 further reduced availability of adenine for purine nucleoside biosynthesis. Second, MTDIA-sensitive gene deletions that were also synthetic lethal with *meu1Δ* included genes involved in gene expression at the levels of chromatin organization, transcription and translation. These results, thus, reinforce the results obtained with the *meu1Δ* genetic mimic of MTDIA and add to our understanding of its mechanisms, namely its involvement with autophagy and lipid metabolism processes.

### High-throughput microscopy identifies changes in protein abundance and localization in response to MTDIA treatment and in *meu1Δ* cells

High-throughput microscopy analysis in yeast measures abundance and localization of proteins within a proteome, and − in the case of drug response − provides insight into the cellular response to a drug (Tkach et al., 2012). To measure abundance and localization of proteins in response to MEU1 deficiency and/or MTDIA treatment, we constructed two new green fluorescence protein (GFP) libraries comprising ∼4900 unique GFP-tagged fusion proteins in the background of dual nuclear and cytosolic red fluorescence protein (RFP) signals with heterologous expression of hENT1. Of those, one library was in a wild-type (WT) background, the other was isogenic with the additional deletion of MEU1. The GFP signals for each protein were quantified relative to the nuclear/cytosolic RFP signals. By using high-throughput confocal microscopy, localization and abundance of individual proteins were assessed in response to MTDIA treatment or in *meu1Δ* cells. Out of ∼4900 GFP/RFP strains grown in three conditions (untreated, MTDIA treated or with expression of *meu1Δ*), 49 proteins were significantly altered regarding their abundance or localization in response to treatment with MTDIA (Table S10).

**Fig. 5.**
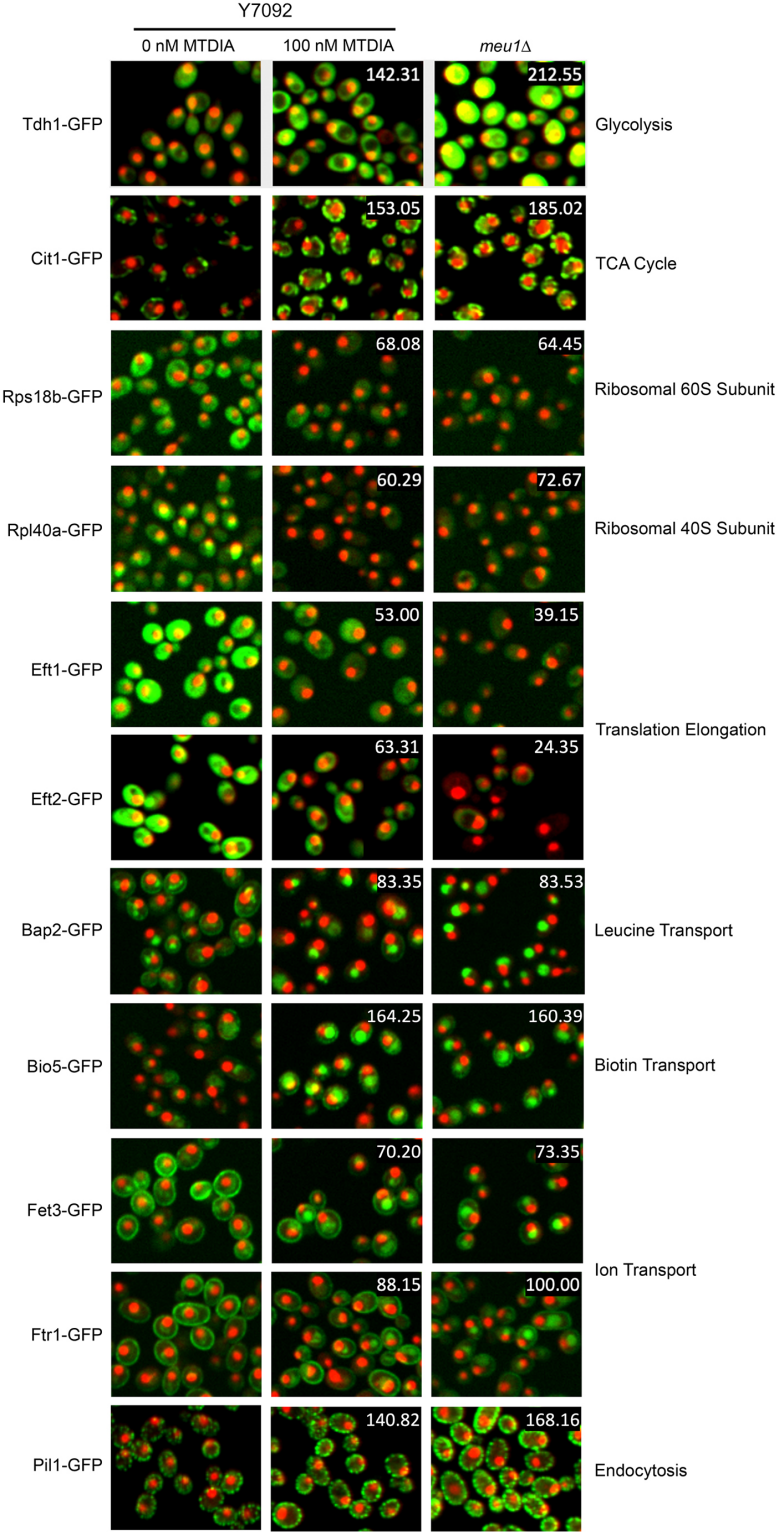
**High-throughput microscopy identifies changes in protein localization and abundance in response to MTDIA and its genetic mimic of MEU1-deficiency.** Representative fluorescence images of proteins involved in specific processes (as indicated) in untreated Y7092 cells, Y7092 cells treated with 100 nM MTDIA and untreated *meu1Δ* cells. Libraries of ∼4900 strains, each expressing a GFP-tagged protein and dual RFP-tagged nuclei/cytoplasm were cultured overnight on SD-U+R agar and inoculated into black-walled, clear-bottom 384-well plates to an OD_660_ of 0.3 in 50 µl volumes of SD-MAU+MTA+RG liquid medium with or without MTDIA. Plates were incubated at 30°C for 6 h and the fluorescent signal was detected at 488 nm (GFP) and 561 nm (RFP). Numbers at top right of middle- and right-column images indicate the percent change in GFP abundance after treatment with MTDIA or in *meu1Δ* relative to untreated Y7092 cells. Changes in protein location were confirmed by visual inspection and validated in independent, reproducible experiments. Cells shown here are representative of three biological replicates each monitoring abundance and localization of 200 cells (*n*=600 cells).

The 49 proteins were classified to seven biological processes. Changes in central carbon metabolism are represented by rate-limiting enzymes in glycolysis (the yeast GAPDH enzyme Tdh1) and the tricarboxylic acid cycle (TCA) cycle (the citrate synthase Cit1) that each were increased after treatment with MTDIA or in *meu1Δ* cells compared to levels in control cells (Fig. 5). In contrast, levels of two main ribosome components (e.g. Rpl40a in the ribosomal 60S subunit and Rps18b in the ribosomal 40S subunit) were each decreased after treatment with MTDIA or in *meu1Δ* compared to those in control cells (Fig. 5). Changes in translation were evident by reduced abundance of two translation elongation factor proteins (Eft1 and Eft2) in response to treatment with MTDIA and in *meu1Δ* cells compared to those in control cells (Fig. 5). Proteins integral to amino acid transport (e.g. Bap2 that transports leucine, and Bio5 that transports biotin) were mislocalized to the vacuole after treatment with MTDIA and in *meu1Δ* cells compared to those in control cells (Fig. 5). Proteins integral to ion transport were also altered after treatment with MTDIA in *meu1Δ* cells. The iron/copper transporter Fet3 mislocalized to the vacuole, and levels of the iron transporter Ftr1 were reduced in the plasma membrane and mislocalized to the vacuole (Fig. 5). Moreover, levels of the eisosome core component Pil1, which marks the initial site of endocytosis at the plasma membrane, were increased after treatment with MTDIA and in *meu1Δ* cells compared to those in control cells (Fig. 5).

### MTDIA impacts lipid metabolism and reduces ergosterol

**Fig. 6.**
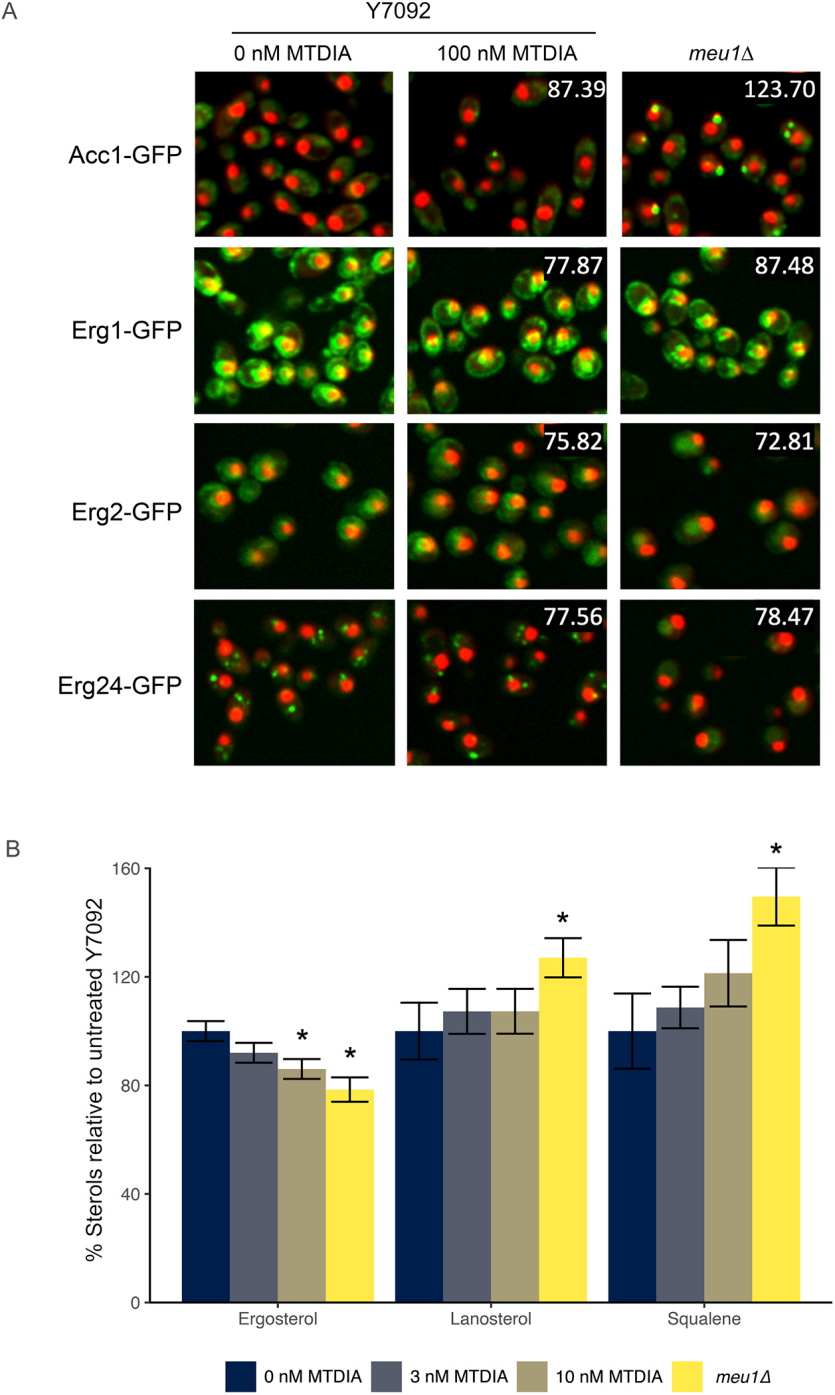
**MTDIA or MEU1-deficiency reduces cellular levels of ergosterol.** (A) Representative fluorescence images of proteins (as indicated) integral to ergosterol biosynthesis untreated Y7092 cells, Y7092 cells treated with 100 nM MTDIA and untreated *meu1Δ* cells. Cells were grown, fluorescence intensity of GFP-tagged proteins and dual RFP-tagged nuclei/cytoplasm was quantified, and localization was assigned as previously described. Numbers at top right of middle- and right-column images indicate the percent change in GFP abundance after treatment with MTDIA or in *meu1Δ* relative to untreated Y7092 cells. Cells shown here are representative of three biological replicates each monitoring abundance and localization of 200 cells (*n*=600 cells). (B) Quantification of sterol intermediates in MTDIA-treated Y7092 or untreated *meu1*Δ cells by using gas chromatography-mass spectrometry (GC-MS). Lipids extracted from 5 OD units of cells grown in SD-MAU+MTA+RG were dried, derivatized, diluted in hexane and quantified using GC-MS. The identity of each sterol was confirmed using authentic standards. The percent (mean±s.d.) of each sterol relative to the total sterols, i.e. ergosterol/(squalene+lanosterol+ergosterol)×100 from six biological replicates is presented. *P<0.05, two-tailed Student's *t*-test comparison with untreated Y7092 cells.

Cholesterol synthesis is upregulated in cancer cells as a means to ensure proliferation (Huang et al., 2020). With the cholesterol synthesis pathway conserved from yeast to humans (Nielsen, 2009), we assessed the impact of MTDIA treatment and the genetic mimic *meu1Δ* on this pathway. Ergosterol is the major sterol in yeast that is synthesized via a 27-step biosynthetic pathway derived from Acetyl-CoA. Both the MTDIA treatment and the genetic mimic *meu1Δ* resulted in the mislocalization of the major Acetyl CoA carboxylase (Acc1) from the cytoplasm to cytoplasmic foci (Fig. 6A). The treatment of MTDIA or *meu1Δ* resulted in the downregulation of enzymes in the middle and late stages of the ergosterol biosynthesis pathway (i.e. the squalene epoxidase Erg1, the C-5 sterol desaturase Erg3 and the C-14 sterol reductase Erg24) (Fig. 6A). Additionally, in the *meu1Δ* strain, Erg3 was mislocalized from the ER to the cytoplasm/vacuole and Erg24 was mislocalized from cytoplasmic foci to the cytoplasm. To further characterize disruptions to sterol biosynthesis pathway, the levels of ergosterol and ergosterol precursors (squalene and lanosterol) were quantified by gas chromatography-mass spectrometry (GC-MS). While reduced levels of ergosterol coincided with increased levels of squalene and lanosterol in *meu1Δ*, the increased treatment dose of MTDIA (10 nM) only reduced ergosterol levels (Fig. 6B). These results suggest that loss of Meu1 activity inhibits ergosterol biosynthesis in yeast.

### Network analysis reveals autophagy as a possible mechanism to buffer MTDIA bioactivity

**Fig. 7.**
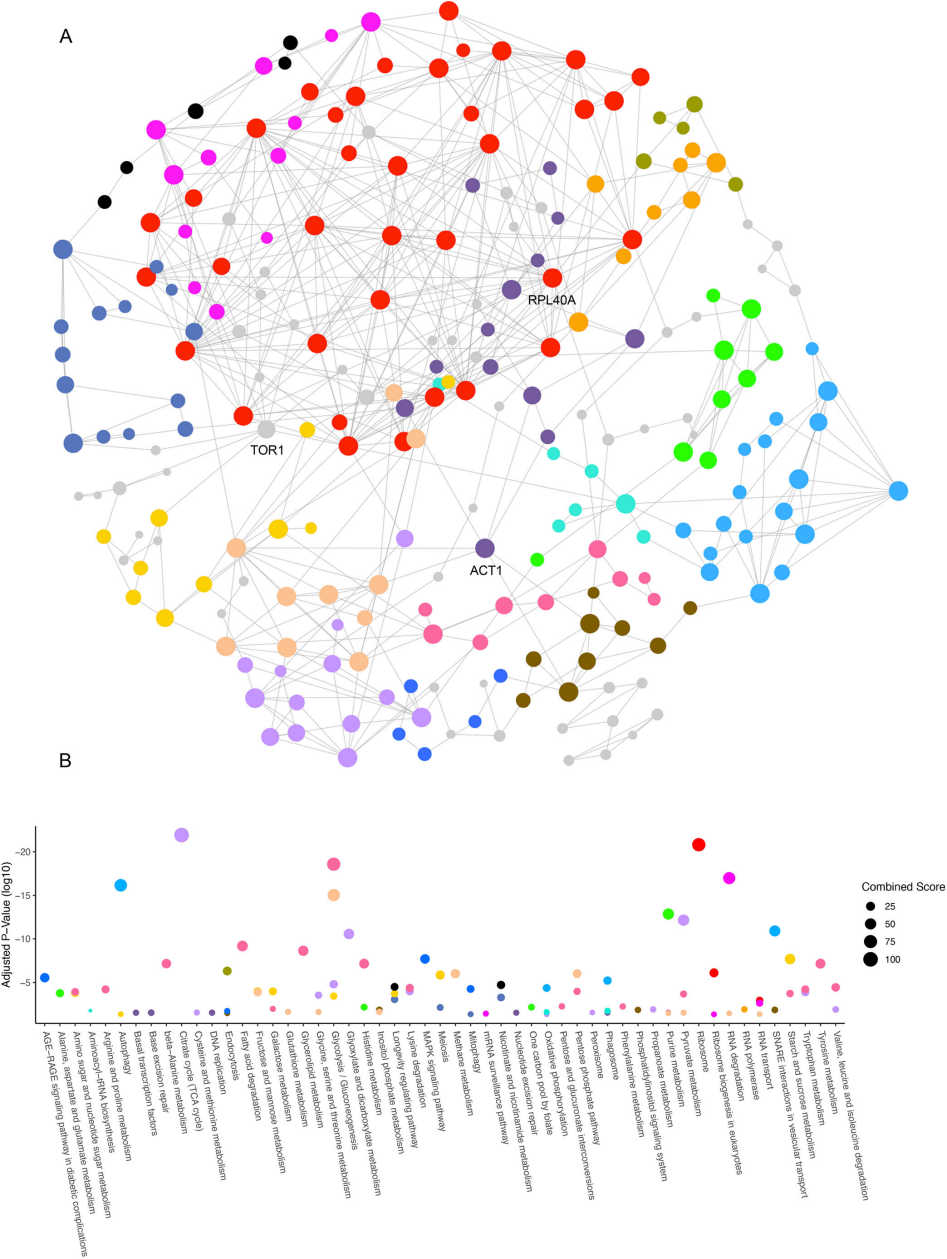
**Network analysis distinguishes autophagy and longevity as enriched pathways in the cellular response to MTDIA.** (A) First-order minimum network analysis of genes/proteins sensitive to MTDIA/*meu1Δ* in growth and microscopy assays. The network was generated in NetworkAnalyst (https://www.networkanalyst.ca/) (Zhou et al., 2019) with the STRING interactome database, where nodes and edges represent genes/proteins and interactions, respectively. Nodes are colored by community analysis (i.e. each community is distinguished in a different color), representing tightly clustered subnetworks with more internal connections than randomly expected in the whole network (*P*<0.05). Nodes not assigned to a community are shown in gray. The most central nodes based on betweenness centrality (TOR1, RPL40A, ACT1) are labeled with their gene name. (B) Pathway enrichment analysis of each community was carried out by comparing the genes/proteins in each community to those in specific pathways in the KEGG database. The size of the bubble reflects the combined score of fold-enrichment and adjusted *P*-value. The color of each bubble is consistent with the communities identified in panel A.

Results, thus far, have been highly informative for annotating the genes and proteins involved in the cellular response to MTDIA. Clearly the response is pleiotropic via multiple pathways and processes. Community analysis of networks can distinguish the most important genes and pathways involved in the cellular response to a drug or disease (Barabási et al., 2011; Busby et al., 2019; Choobdar et al., 2019). The most important genes can be identified by betweenness centrality, a quantitative value for every gene in a network that reflects the importance of that gene to the integrity of the network. Pathways can be identified via pathway enrichment of communities in the network. Network analysis was, thus, conducted to account for genes, proteins and their associated pathways interacting with the genes. By using NetworkAnalyst (https://www.networkanalyst.ca) (Zhou et al., 2019), the 185 genes/proteins identified above as being sensitive to treatment with MTDIA or in *meu1Δ* cells (hereafter referred to as MTDIA/*meu1Δ*) in growth- and microscopy assays were mapped within an established global network comprising the interactions in the STRING interactome database (https://string-db.org) (Szklarczyk et al., 2015). This first-order network comprising only the 185 genes/proteins and their interactors was then trimmed to a minimum network to keep only the nodes necessary to connect genes/proteins sensitive in cells treated with MTDIA or in *meu1Δ* cells (Fig. 7A). The genes most highly ranked in betweenness centrality (Table S11) were the major autophagy-regulating TOR1 kinase (5148.87), the ribosomal 60S subunit RPL40A (4296.51) and the cytoskeletal actin gene ACT1 (4145.48), reflecting the importance of protein/cargo homeostasis as a cellular response to MTDIA. By using the InfoMap algorithm, 14 statistically significant communities were identified in the network. To evaluate over-representation of pathways in these modules, an enrichment analysis of interactions for association to the KEGG pathway database distinguished 53 pathways critical to the cellular response to MTDIA (Fig. 7B). Notably, the processes associated with the three top betweenness genes are therapeutic targets to treat cancer (i.e. autophagy, the ribosome and the cytoskeleton) (Debnath et al., 2023; Pelletier et al., 2018; Suresh and Diaz, 2021), and our protein abundance analysis above experimentally validates the importance of Rpl40A to the mechanism of MTDIA (Fig. 5).

### MTDIA exacerbates rapamycin-induced growth arrest and induces autophagic cell death

**Fig. 8.**
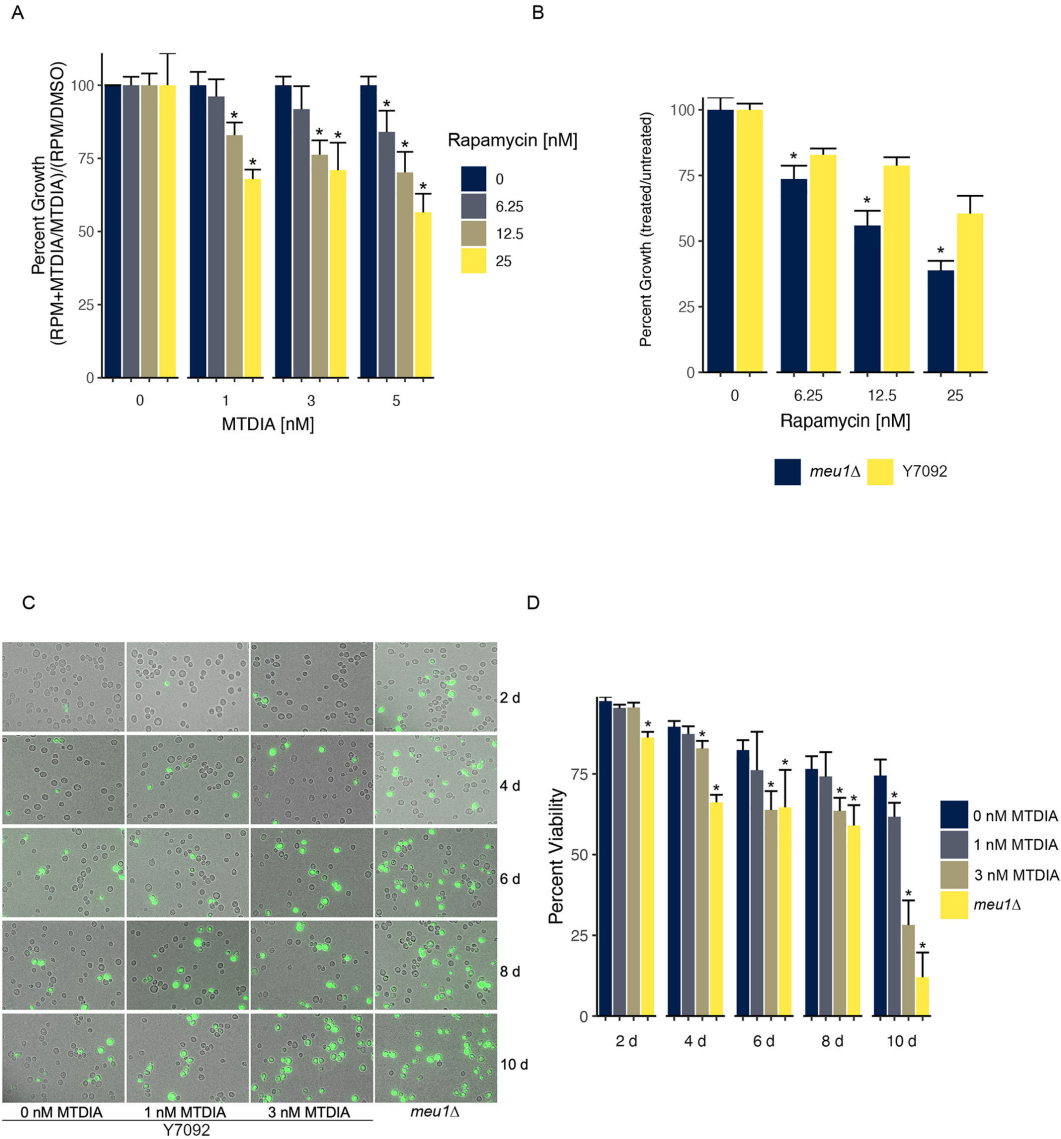
**MTDIA treatment and *meu1Δ* exacerbates the growth defect induced by rapamycin and decreases chronological lifespan.** (A) Growth of Y7092 cells treated with rapamycin (RPM) and MTDIA as indicated relative to MTDIA treatment alone. Cells were grown at 30°C and growth was quantified at 48 h via OD_600_ readings. Percent growth was determined via growth in the liquid from the growth in the MTDIA+RPM co-treatment relative to MTDIA or RPM alone [(RPM+MTA/MTDIA)/(RPM/DMSO)×100] from six biological replicates. *P<0.05, two-tailed Student's *t*-test comparison of treated and untreated cells. (B) Sensitivity of *meu1Δ* cells to RPM is enhanced compared to that of Y7092 cells. Cells were grown at 30°C and growth was quantified at 48 h via OD_600_ readings. *P<0.05, two-tailed Student's *t*-test comparison of Y7092 and *meu1Δ* cells at each RPM concentration for six biological replicates. (C) Representative fluorescence images of MTDIA-treated Y7092 and untreated *meu1Δ* cells in a chronological lifespan assay. Cells were cultured in 50 ml of SD-MAUC+MTA+RG medium at 30°C for 10 days. At 2-day intervals, 1 ml of cells was stained with 2 µg/ml Phloxine B to distinguish dead cells and visualized at 60× magnification under DIC and GFP filter with a fluorescent microscope (Olympus BX63) and a digital camera (Olympus Dp70) (*n*=1000 cells). (D) Quantification of viability based on Phloxine B fluorescence at 2-day intervals. Percent viability [(unlabeled cells/GFP labelled cells+unlabelled cells)×100] and standard deviation were determined from 1000 cells across five visual fields for each condition. *P<0.05, two-tailed Student's *t*-test comparing MTDIA-treated Y7092 or untreated *meu1Δ* cells with untreated Y7092 cells at each time point. Error bars indicate the mean+s.d.

Several lines of evidence described above, such as enriched growth-sensitivity of gene deletions involved in autophagy (Table S4), two network modules enriched in autophagy (Fig. 7B), and a major regulator of autophagy (TOR1) as a the top-ranked betweenness centrality gene (Fig. 7A) in a network analysis, point to autophagy as a potential novel mechanism of action for MTDIA. To determine if MTDIA treatment disrupts the autophagic flux, cells were co-treated with MTDIA and rapamycin, an inhibitor of the TOR nutrient sensing pathway and a hallmark inducer of autophagy (Kamada et al., 2000). The growth of Y7092 cells treated with semi-inhibitory concentrations of MTDIA or its genetic mimic *meu1Δ* were assessed following treatment with rapamycin (Fig. 8A). A concentration-dependent growth reduction of Y7092 cells was observed following treatment with rapamycin (6.25, 12.5 or 25 nM) and exacerbated by treatment with 1, 3 or 5 nM MTDIA. This increased sensitivity to rapamycin was also observed for *meu1Δ* compared to Y7092 cells (Fig. 8B). However, the *meu1Δ* strain was slow growing as it is unable to utilize MTA and relied on other methionine-salvage pathways for growth (Table S9). These results are also consistent with the *TOR1* gene deletion being sensitive to MTDIA (Table S5). Together, these results demonstrate that levels of MTDIA as low as 1 nM are required for rapamycin-induced growth arrest, suggesting that autophagy is a mechanism of MTDIA toxicity.

Measurement of cell survival is one approach to monitoring the impact of autophagy; this can be assayed with Phloxine B, which stains the cytosol of dead cells comprising disrupted plasma membranes (Klionsky et al., 2021). To investigate autophagic cell death, Y7092 cells treated with MTDIA and untreated *meu1Δ* cells were assessed for viability with Phloxine B (Fig. 8C,D). Y7092 cells treated with MTDIA showed a reduction in viability starting 4 days after treatment [82.9% for 3 nM MTDIA (∼IC_20_) versus 89.6% for untreated Y7092 cells]. This defect was further enhanced at 10 days after treatment, resulting in a concentration-dependent reduction in viability after treatment with MTDIA [61.7% for 1 nM MTDIA (∼IC_10_) and 28.2% for 3 nM MTDIA versus 74.5% for untreated Y7092 cells]. The decrease in cell viability was further exacerbated in untreated *meu1Δ* cells (66.2% at 4 days versus 13.0% at 10 days). These results showed that MTDIA-dependent MTAP inhibition over several days is cytotoxic; moreover, treatment with MTDIA induced toxic levels of autophagy in yeast. This is consistent with the cytotoxic treatments at concentrations >0.316 µM MTDIA (Fig. 3C).

### MTDIA regulates abundance and localization of core autophagy proteins

**Fig. 9.**
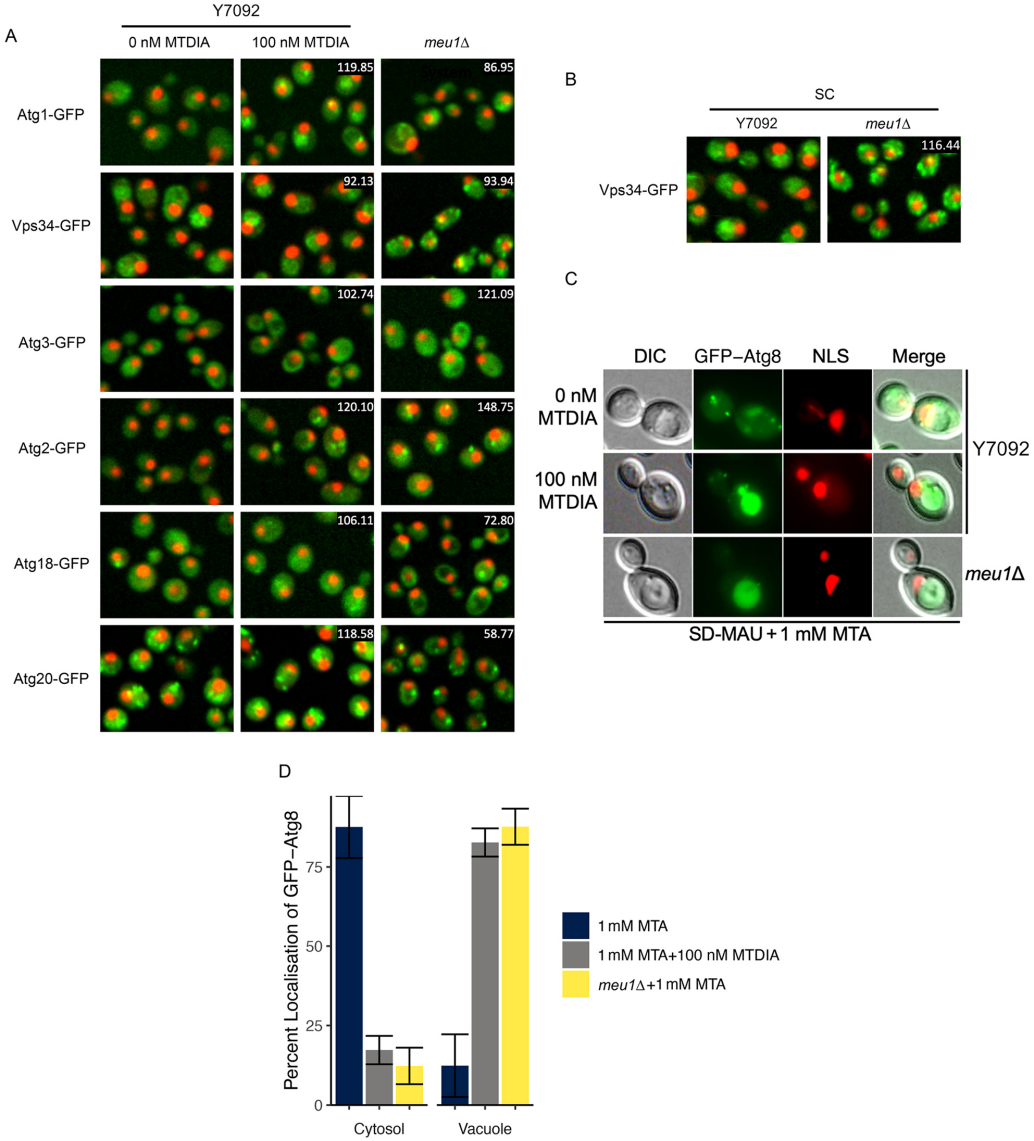
**MTDIA treatment or *meu1Δ* induces autophagy independent of starvation.** (A) Fluorescence images showing abundance and localization of GFP/RFP-tagged autophagy proteins in untreated Y7092 cells, Y7092 cells treated with 100 nM MTDIA and untreated *meu1Δ* cells. Cells were grown in SD-MAUC+MTA+RG medium for 6 h at 30°C, fluorescence was quantified, and localization was assigned as previously described. Numbers at top right of middle- and right-column images indicate the percent change in GFP abundance in Y7092 cells after treatment with MTDIA or in *meu1Δ* cells relative to untreated Y7092 cells. (B) Fluorescence images showing abundance and localization of Vps34-GFP/RFP in Y7092 or *meu1Δ* cells. Cells were grown in synthetic complete medium supplemented with 5′-methylthioadenosine (SC+MTA) for 6 h at 30°C, GFP fluorescence was quantified and protein localization assigned as described in Materials and Methods. Number inset in the right image indicates the percent changes in GFP abundance in *meu1Δ* compared to Y7092. (C,D) Representative images (C) and quantification of fluorescence intensity (D) of GFP-Atg8 in the cytosol or vacuole of MTA-treated Y7092, MTA/MTDIA-treated Y7092 or MTA-treated *meu1Δ* cells. NLS indicates staining of nuclei. Error bars indicate the mean±s.d. Cells shown in panels A-C are representative of three biological replicates and 600 cells.

The autophagy pathway in yeast has been extensively studied and genes involved in each step of autophagy: induction, i.e. recognition and packaging of cargo, vesicle nucleation, expansion of the phagophore and autophagosome completion, cycling of autophagy (Atg) proteins, autophagosome fusion with the vacuole, degradation of cargo and recycling of macromolecules have been identified (Ohsumi et al., 2009). To further investigate the induction of autophagy in response to MTDIA treatment, we measured abundance and localization of core autophagy proteins in response to MTDIA treatment and MEU1-deficiency (Fig. 9A). The autophagy proteins Atg1 (Atg1 kinase complex), Vps34 (phosphoinositide 3-kinase), Atg3 and Atg4 (Atg8 conjugation system), Atg2 and Atg18 (Atg9 recycling), and Atg20 (cytoplasm-to-vacuole targeting pathway) showed changes in abundance and/or localization in response to MTDIA treatment and in *meu1Δ* cells. Atg1-GFP relocalized from endosomes to the vacuole and, similarly, Atg2-GFP relocalized from the cytosol to endosomes in cells treated with MTDIA and in *meu1Δ* cells, which would be consistent with rapamycin-induced autophagy phenotypes. Atg3-GFP, Atg18-GFP and Atg20-GFP were each excluded from the vacuole in *meu1Δ* cells, which likely reflected nutrient starvation with total loss of Meu1. Likewise, Vps34-GFP showed a change in localization from the vacuole and vacuolar membrane to cytoplasmic foci and the ER in *meu1Δ* cells but not in cells treated with MTDIA. Interestingly, as in Y7092 cells, mislocalization of Vps34 in *meu1Δ* cells was also observed in complete minimal (methionine-replete) medium (Fig. 9B). This change in Vps34 localization in complete medium may present a likely point of perturbation for autophagy induction that is independent of amino acid starvation.

### MTDIA induces autophagic trafficking of GFP-Atg8 independent of starvation

To further confirm MTDIA induces autophagy, the localization of GFP-Atg8, the yeast orthologue of the mammalian autophagosome marker LC3, was evaluated. LC3/Atg8 is one of two proteins that remain associated with the autophagosome throughout the autophagy pathway, and is frequently used as a marker for monitoring autophagic activity in yeast and mammalian cells (Klionsky et al., 2021). Following induction of autophagy in starvation medium or following rapamycin treatment, Atg8 localizes from cytosolic foci (autophagosomes) to the vacuole (Ohsumi et al., 2009). The localization of GFP-Atg8 in Y7092 and *meu1Δ* cells was assessed by fluorescence microscopy after incubation in MTA-containing medium supplemented with and without MTDIA (Fig. 9C,D). In the absence of MTDIA, ∼88% of the Y7092 cells showed cytosolic location for GFP-Atg8. There was no evidence of autophagy in medium without MTDIA, which demonstrates that medium alone that lacks a few amino acids, is not inducing starvation-induced autophagy. Following 100 nM MTDIA treatment for only 6 h, the direct opposite was observed, i.e. nearly 90% of the cells showed vacuolar localization of GFP-Atg8. This autophagy-inducing phenotype was conserved in the *meu1Δ* strain in the absence of MTDIA, wherein vacuolar localization of GFP-Atg8 was observed. Overall, increased localization of GFP-Atg8 to the vacuole following treatment with MTDIA, which is dependent on a functional autophagic pathway, confirms an induction of autophagy as a main mechanism of action of this drug.

## DISCUSSION

We have elucidated mechanisms of action for the transition-state structure analogue MTDIA, a potent inhibitor of MTAP. It is necessary to know the compendium of cellular perturbations in response to this drug that demonstrated efficacy *in vivo* and *in vitro* against lung, prostate, colon, cervical and triple-negative breast cancer. Genomic analyses of our newly developed yeast system expressing hENT1 for nucleoside uptake permitted an unbiased genome-wide assessment of the cellular effects of MTAP inhibition in response to (i) MTDIA treatment and, (ii) MTAP deletion by knocking out the yeast MTAP gene MEU1. Either set-up was investigated by using gene deletion and protein localization/abundance analyses to provide complementary perspectives to identify genes and proteins integral to these conditions. Our research highlights gene−gene and gene−drug interactions discerned with MTDIA treatment or its genetic mimic *meu1*Δ, demonstrating that even highly target-specific inhibitors affect large numbers of interacting genes. Of these, many are involved in fundamental processes beyond the initiating apoptotic pathways (Basu et al., 2007) and changing DNA methylation patterns (Basu et al., 2011) that are known mechanisms of MTDIA.

MTDIA is known to reduce polyamine levels, induce methionine deficiency and alter the chromatin/histone status (Basu et al., 2007, 2011; Firestone et al., 2021). Based on the conserved processing of MTA by MTAP/Meu1 that is necessary to salvage methionine from yeast to humans (Pirkov et al., 2008), we developed yeast models were developed recapitulating these mechanisms. Most gene deletions sensitive to *meu1*Δ or MTDIA were involved in chromatin organization, transcription and translation, consistent with findings in mammalian cell lines and murine models (Basu et al., 2007, 2011; Mandal et al., 2013; Pollard et al., 1999). Moreover, since yeast does not encode DNA methyltransferases, the mechanisms by which MEU1 affects chromatin organization are independent of S-adenosylmethionine-dependent reactions. Reduced sensitivity of *meu1*Δ*spe3*Δ was detected for the combinatorial effect of these enzymes, critical to methionine and polyamine metabolism.

Autophagy is an intracellular degradation pathway where cytosolic content is degraded via vacuoles and recycled to compensate for nutrient deprivation (Suzuki and Ohsumi, 2010). Autophagy has not been reported as a mechanism for MTDIA, and was only investigated here because of results with unbiased genomic and proteomic analyses. Given the strong connection between amino acid (specifically methionine) deficiency activating autophagy, our identification of autophagy as a mechanism for MTDIA function is reasonable and unlikely to be limited to the yeast models used in this study. Based on our observations that MTDIA treatment or *meu1*Δ dramatically impacts fundamental aspects of central carbon metabolism as well as inducing autophagy, it is plausible that autophagy is activated via a mechanism involving acetyl-CoA − particularly since acetyl-CoA is an established regulator of autophagy (Mariño et al., 2014) and major regulators of Acetyl-CoA were altered after treatment with MTDIA and in *meu1Δ* cells. Intriguingly, acetyl-CoA levels are elevated in various cancers by upregulation of enzymes that metabolize acetate, such as acetyl-coA synthetases (Comerford et al., 2014). Moreover, downregulation of this enzyme reduced the tumor burden in models of hepatocellular carcinoma (Comerford et al., 2014) and acetyl-CoA transferase ACAT1 was downregulated in A549 tumors following treatment with MTDIA (Basu et al., 2011), and the yeast orthologues were downregulated in *meu1*Δ cells. Given that rapamycin is a candidate anti-cancer drug and we were able to show that MTDIA enhanced activity of rapamycin, it is worthwhile to investigate a dual treatment strategy by using rapamycin and MTDIA in human cancer cell lines.

Acetylation of the phosphoinositide 3-kinase Vps34 by acetyl-CoA regulates autophagy through conversion of phosphoinositide to phosphoinositide 3-kinase, and is required for autophagosome formation, maturation and transport (Dall'Armi et al., 2013). Vps34 activates TOR1 in yeast (mTOR in mammalian cells) in the presence − but not absence − of glutamine and, therefore, plays a role in starvation-induced autophagy (Tanigawa and Maeda, 2017). Vps34 relocalizes from vacuoles/lysosomes to subdomains of the ER upon starvation (Burman and Ktistakis, 2010) and exogenously added phosphoinositide 3-kinase induces autophagy in human colon cancer cells (Petiot et al., 2000). Therefore, our observation that Vps34 is relocalizing from vacuoles to − what appears to be – subdomains of the ER in *meu1*Δ cells under methionine-replete conditions, suggests that Vps34 is the inducer of autophagy. Interestingly, Vps34 inhibitors (e.g. alpelisib and buparlisib) are promising anti-cancer drugs currently in clinical trials (Castel et al., 2021); thus, our results indicate synergy with MTDIA could be an effective dual therapy.

Likewise, our result that treatment with MTDIA inhibits sterol synthesis suggests that MTDIA in combination with sterol-inhibiting statins may improve the anti-cancer activity of both compounds. Statins are currently undergoing or have recently completed hundreds of clinical trials for its anti-cancer efficacy against various cancers, including lung, liver, breast and prostate cancer (Huang et al., 2020). Consistent with our study was the fact that MTDIA reduces levels of esterified ergosterol in yeast (Salita et al., 2023) and that MTDIA treatment of A549 tumors causes a compensatory more than threefold increase in the expression of cholesterol homeostasis genes (Basu et al., 2011; Kee et al., 2004). Inhibition of sterol biosynthesis in yeast or mammalian cells may be mediated through inhibition of MTAP/Meu1, leading to accumulation of decarboxylated S-adenosyl methionine, an inhibitor of S-adenosylmethionine and a coenzyme required for sterol synthesis (Frostesjö et al., 1997; McCammon et al., 1984). Since statins also induce autophagy and are, at least in part, responsible for the anti-metastatic effects of statins (Parikh et al., 2010), it is plausible that autophagy is linked with the anti-cancer mechanisms of MTDIA, being mediated through disruptions to lipid homeostasis in a manner similar to that of statins. The changes in lipid metabolism by MTDIA (Salita et al., 2023) might induce other selective forms of autophagy, such as mitophagy, predicted by our network analysis.

In conclusion, the results of this study provide a global survey of the cellular effects of MTAP inhibition, either in response to treatment with MTDIA or following MEU1 deletion. Suspected pathways were identified that justify the use of yeast, and notable among the unsuspected pathways was the induction of autophagy via a rapamycin-independent and Vps34/PI3P-dependent mechanism. As these mechanisms are conserved from yeast to humans, MTAP inhibition might also induce autophagy in humans and be a mechanism for MTDIA-mediated death of cancer cells. Given that small molecules modulating autophagy in a myriad of human disorders − including cancer, heart disease, obesity and neurodegeneration − are the focus of intensive investigation (Jiang and Mizushima, 2014; Kovsan et al., 2011; Levine and Kroemer, 2008), the results herein suggest that MTDIA can be considered among these autophagy modulators and possibly as a therapeutic treatment for diseases other than cancer. In addition to clinical trials investigating the efficacy of MTDIA alone in cancer mouse models that have effectively been treated with MTDIA (Basu et al., 2007, 2011; Firestone et al., 2021), our results indicate that a synthetic lethal approach is also effective (e.g. synergy of MTDIA and rapamycin), particularly with 15% of all cancers being classified as ‘MTAP cancers’, i.e. as cancers comprising upregulated levels of MTAP.

## MATERIALS AND METHODS

### Chemicals

MTDIA was synthesized as previously described (Basu et al., 2007), dissolved in DMSO at a concentration of 10 mM and stored at −20°C. MTA (AK Scientific) was dissolved in ddH_2_O at a concentration of 5 mM and stored at −20°C.

### Strains

All *S. cerevisiae* strains for MEU1-interaction analyses were constructed by PCR-mediated disruption and synthetic genetic array (SGA) analysis in the background of the Y7092 strain (Table S12), with the addition of heterologous expression of human equilibrative nucleoside transporter 1 (hENT1) gene from plasmid pYhENT1 (Vickers et al., 1999).

### Media

All *S. cerevisiae* strains were cultured in yeast peptone dextrose (YPD), synthetic complete (SC) or complete minimal (CM) medium supplemented with 100 µg/ml ClonNAT (Werner bioAgents) and/or 200 µg/ml geneticin (G418) (Table S13). The amino acid analogues L-canavanine (Sigma-Aldrich) and L-thialysine (Sigma-Aldrich) were used at 50 µg/ml in the absence of their amino acid analogs. All media were autoclaved at 121°C and cooled to 65°C before adding the carbon source (2% glucose, 2% raffinose or 2% galactose) and antibiotics. All *E. coli* strains were cultured in lysogeny broth (LB) supplemented with 100 µg/ml ampicillin (Sigma-Aldrich).

### Liquid growth assay

All liquid growth assays were performed in 96-well plates (Jet Biofil) as previously described (Busby et al., 2019). Cells were grown in liquid overnight without treatment, subcultured to 5×10^5^ cells/ml, treated with either compound or vehicle control, incubated at 30°C, and absorbance (OD_600_) measured hourly using the Envision Xcite Multilabel Reader (Perkin-Elmer). Growth was quantified by calculating the percentage of residual growth ((OD_600 treatment_/OD_600 vehicle control_)×100) at a time point when control cells were at mid-log (OD_600_=0.3−0.5).

### Agar growth assay

All agar growth assays were performed in 24-well plates (Jet Biofil) as previously described (Busby et al., 2019). Cells were grown in liquid overnight without treatment, diluted to three concentrations (1×10^5^, 1×10^3^ and 1×10^1^ cells), plated on agar for each condition of compound or vehicle control, incubated at 30°C, and imaged with a digital camera at 24 h and 48 h in SC+glucose medium or 48 h and 96 h in SD-MAUC+2% raffinose+2% galactose medium.

### Nucleoside uptake

To engineer the uptake of nucleosides, yeast strains were transformed with either the human equilibrative nucleoside transporter (hENT1) (Vickers et al., 1999) or empty vector pYES2. The *ade2Δade3Δ* double mutant was cultured in media supplemented with MTA or adenosine in the absence of methionine or adenine, respectively. Bioactivity of known toxic nucleoside analogues 3′-deoxyadenosine (cordycepin) and 7-deaza-7-cyanoadenosine (toyocamycin) was evaluated by using the aforementioned liquid growth assay.

### Analysis of genetic interaction with MEU1

To investigate genetic interactions with MEU1 (the yeast equivalent of human MTAP), a genome-wide SGA analysis (Tong et al., 2001) was conducted using a query strain in the Y7092 background with the addition of meu1::NATR introduced by PCR-mediated gene disruption. Growth of each *meu1ΔxxxΔ* double mutant on SD-MAUC+MTA+RG was quantified using Gitter (Wagih and Parts, 2014) and ScreenMill (Dittmar et al., 2010). Double-gene deletion strains that showed a growth defect (growth<40%, *P*<0.05) or growth improvement (growth>40%, *P*<0.05) compared to the haploid single-deletion parents in at least two out of three SGAs were considered as putative genetic (epistatic) interactions of MEU1. These genes as well as functionally related genes were independently validated via an additional SGA and quantification of growth in the aforementioned liquid growth assay. The epistatic growth defect of the double mutants was calculated relative to the growth defect of the *meu1Δ* single mutant [(*meu1ΔxxxΔ*/*meu1Δ*)/(*xxxΔ*/WT)], modified from the calculation used for assessment of drug sensitivity. The relative growth of the double mutants to the single mutants in SD-MAUC+MTA+RG was normalized against the *meu1Δhis3Δ* double-gene deletion strain, which is phenotypically identical to *meu1Δ* single-gene deletion strain that contains a partial deletion of the HIS3 gene. Stringent cut-offs of growth reduction (40% treated compared to untreated with *P*<0.05) were considered synthetic lethal with the *meu1Δ* strain.

### MTDIA toxicity

MTDIA toxicity was evaluated by growing MTDIA-treated cells on MTDIA-free medium. Y7092+hENT1 cells were incubated at 30°C in 100 µl of SD-MAUC+MTA+2% raffinose+2% galactose (SD-MAUC+MTA+RG) medium treated with inhibitory concentrations of MTDIA, and 1 µl of cells was removed at 0, 6, 24 and 48 h. Cells were then plated on SD-MAUC+MTA or SD-U agar media, incubated at 30°C and imaged at 48 h using a digital camera (Canon EOS 600D).

### Construction of a human ENT1 (hENT1) yeast gene deletion library for chemical genetics interaction analysis with MTDIA

To construct the haploid yeast gene deletion library expressing hENT1 (*xxxΔ*+hENT1), the Y7092+hENT1 strain was crossed with the non-essential haploid yeast gene deletion library using SGA methodology (Tong et al., 2001). The resulting haploid *xxxΔ*+hENT1 gene deletion library was freshly pinned in 1536 format on SD-U+4% raffinose agar plates and incubated at 30°C for 24 h. Colonies were then replicated on SD-MAUC+MTA+RG agar plates with and without MTDIA, incubated for 48 h at 30°C, and photographed using a digital camera (Canon EOS 600D). Growth of each *xxxΔ*+hENT1 mutant was quantified using Gitter (Wagih and Parts, 2014) and ScreenMill (Dittmar et al., 2010). Strains that showed a growth defect (growth<40%, *P*<0.05) or growth improvement (growth>40%, *P*<0.05) with MTDIA treatment compared to untreated cells in at least two out of three SGAs were considered as putative epistatic interaction of MEU1, and these genes as well as functionally related genes were independently validated via an additional SGA and quantification of growth in the aforementioned liquid growth assay.

### Construction of human ENT1 (hENT1) and MEU1-deficient yeast GFP strains for proteomic analysis

Libraries of strains expressing a C-terminal GFP unique to each protein, and common dual RFP markers for the nucleus (NLS-RedStar2) and cytoplasm (TEF2pr_mCherry) were constructed using SGA analysis and PCR-mediated disruption. First, the MATa GFP library (Huh et al., 2003) was crossed with a MATα query strain expressing hENT1 via a URA3 selectable marker and a single selectable marker (LEU2) for the dual RFPs. The CAN1 and LYP1 loci in BY4741 were replaced with STE2pr-Sp_LEU2 from Y7039 and the HPH::TEFpr_NLS-RS2::TEF2pr_mCherry construct from YCG383. BY4742 was transformed with pYhENT1 (CEN3::hENT1::URA3) (Janke et al., 2004; Vickers et al., 1999). The resulting BY4741-based strain (can1Δ lyp1Δ::HPH::TEFpr_NLSRS2::TEF2pr_mCherry) and the BY4742+hENT1::URA3 transformants were mated to generate a diploid strain, which was then cultured overnight in SD-U+HPH+ 2% glucose liquid medium, washed with water, resuspended in sporulation media, incubated at 30°C for 7 days, plated on SD-ALU+CT+2% glucose agar, and incubated for 2 days at 30°C. The resulting colonies were inoculated in SD-U medium in a 96-well plate (one colony per well) and incubated at 30°C overnight. Random spore analysis was performed in liquid media, where ∼1 µl of spores in sporulation medium was cultured in SD-U medium and transferred into SD-L, SD-MC, SD-H, SD-U or SD-MAUC+2% galactose liquid media to genotype strains. Concomitantly, MATa (his3::kanR) and Matα (pdr1::natR) mating-type strains with selectable markers were cultured overnight, resuspended in YPD to an OD_600_ of 0.3 in 90 µl of either MATa or MATα. Cells were then transferred into 96-well plates followed by 10 µl SD-U cultured spores and incubated at 30°C for 1 day. Then 10 µl of cells (mated with MATa or MATα) were inoculated into SD-U+G418 or SD-U+NAT and incubated at 30°C for 2 days. Eight spores were acquired by random spore analysis that showed phenotypes consistent with WT YCG640 genotype (MATα can1Δ::STE2pr-Sp_LEU2; lyp1Δ::HPH::TEFpr_NLS-RS2::TEF2pr_mCherry; his3Δ1 leu2Δ0 ura3Δ0 met15Δ0 LYS2 CEN3::hENT1::URA3). Of these, one was used as query strain in the construction of the GFP/dual RFP+hENT1 strains by SGA methodology as previously described (Tong et al., 2001). Subsequently, in the resulting library, MEU1 was replaced by PCR-mediated disruption with pFA6-kanMX4 to generate a query strain in SGA analysis to construct the GFP/dual RFP+hENT1 strains in the *meu1Δ* background.

### Automated fluorescence microscopy

High-throughput microscopy analysis was conducted as previously described (Bircham et al., 2011), with additional specifications for the newly constructed libraries. Cells expressing the GFP-tagged protein and dual RFPs were maintained in 384 colony format on SD-U+2% raffinose agar, cultured at 30°C for 20 h, and inoculated into a black-walled clear bottom 384 well cell carrier plate (Perkin Elmer) to an OD_660_ of 0.3 in 50 µl volumes of SD-MAU+MTA+2% galactose liquid medium with and without MTDIA. Plates were incubated at 30°C for 6 h, the fluorescent signal was detected at 488 nm (GFP) and 561 nm (nuclear localization signal fused to RedStar2 and cytosolic mCherry) using the 60× water immersion lens (NA 1.2) in the high-throughput spinning disk confocal microscope (Evo Tec OPERA, Perkin Elmer). Three visual fields were imaged for each condition. Changes in GFP intensity (>20% increase or decrease compared to untreated) were quantified using Acapella automated image analysis software (Perkin Elmer). Changes in localization were confirmed by visual inspection and validated in independent, reproducible experiments. A cut-off >20% change in abundance was defined as significant, as this was consistently detectable by visual inspection.

### Gas chromatography-mass spectrometry analysis of sterols

Ergosterol, lanosterol and squalene were quantified in cells by using gas chromatography-mass spectrometry (GC-MS) as previously described (Munkacsi et al., 2011). Strains were grown at 30°C in SD-MAUC+250 μM MTA+2% galactose medium with and without MTDIA, and lipids were extracted from 5 OD units. Dried lipids were derivatized with 1.2 mg/ml methoxamine hydrochloride in pyridine at room temperature for 16 h, followed by silylation with 30 μl of N-methyl-N-(trimethylsilyl) trifluoroacetamide with 1% trimethylchlorosilane at 70°C for 30 min. Data acquisition was performed on a Shimadzu QP2010-Plus gas chromatograph-mass spectrometer (Shimadzu) and data analysis was performed using Shimadzu GC-MS solution v2.72 (Shimadzu). Sterols were identified by comparison of detected fragmentation patterns and retention times with those of authentic standards for ergosterol, lanosterol and squalene (Avanti Polar Lipids).

### Enrichment analysis

Over-representation of genes and proteins sensitive to MEU1-deficiency and MTDIA treatment for specific functions, processes, components and pathways was investigated using YeastEnrichr (Kuleshov et al., 2016). Significant enrichment was defined for categories with an adjusted *P*-value of <0.05, whereby the *z*-score (ratio of expected and observed term) and combined score (the product of the *z*-score and negative logarithm of the *P*-value) in tandem with the identity of the genes in each category provided full annotation of the enriched categories.

### Network analysis

Genes and proteins sensitive to MEU1-deficiency and MTDIA treatment were annotated for function via network- and pathway-based community partitioning as previously described (Busby et al., 2019). By using NetworkAnalyst (Zhou et al., 2019), genes and proteins were mapped in a first-order network within an established yeast global genetic-interaction network − specifically the STRING interactome (Szklarczyk et al., 2015) − with a confidence cut-off of 900 and requirement for experimental evidence. Significant community modules (*P*<0.05) were identified by using the InfoMap algorithm, and pathways enriched in these modules were determined using KEGG pathway analysis (FDR<0.05).

### Intracellular trafficking analysis of autophagy

Autophagy was monitored via intracellular trafficking of GFP-Atg8 as previously described (Klionsky et al., 2021). Cells were transformed with GFP-Atg8 plasmid and an overnight culture of a transformant was subcultured to OD_660_=0.2, incubated in SD-U+2% raffinose overnight at 30°C, washed twice in dH_2_O, resuspended in fresh medium containing glucose or galactose with or without MTDIA, and cultured at 30°C for 3 h. Cells expressing GFP-Atg8 were visualized at 100× magnification using a fluorescent microscope (Olympus BX63) equipped with a digital camera (Olympus Dp70).

### Viability analysis of autophagy

Autophagy was monitored through viability of cells stained with Phloxine B (Sigma-Aldrich) as previously described (Klionsky et al., 2021). Cells (OD_660_=0.2 for WT, 0.6 for *meu1Δ* and 10 nM MTDIA) were cultured overnight in SD-U+2% raffinose, washed twice with 10 ml CM-MAUC+RG+MTA, resuspended in 50 ml of CM-MAUC+RG+MTA and cultured for 10 days at 30°C. At 0, 2, 4, 6, 8 and 10 days, 1 ml of culture was transferred into a microfuge tube, cells were stained with 2 µg/ml Phloxine B and visualized at 60× magnification using a fluorescent microscope (Olympus BX63) equipped with a digital camera (Olympus Dp70). Percent viability was calculated [(unlabeled cells/GFP labelled cells+unlabelled cells)×100] by using ≥1000 cells across five visual fields for each condition.

## Supplementary Material

10.1242/dmm.052173_sup1 Supplementary information

Table S1. Annotation of the genetic interactions that confer reduced growth or increased growth of meu1Δ. The growth of the double mutants relative to the single haploid parental strains (*meu1ΔxxxΔ/meu1Δ*)/(*xxxΔ/WT*) × 100 was determined at a time point when the untreated *xxxΔ* cells were at mid-log. Relative to the parental *meu1Δ* and *xxxΔ* single gene deletion strains, the double deletion mutants with an average growth <75% (*P*<0.05) or >130% (*P*<0.05) were considered a growth defect or a growth improvement, respectively. Function/process is derived from *Saccharomyces* Genome Database.

Table S2. The validated gene deletions sensitive to *meu1Δ* were assessed for enrichment to the major hierarchical gene ontology (GO) category Biological Process using YeastEnrichr with *P*-values corrected for multiple testing using the Benjamini -Hochberg false discovery rate of 0.05.

Table S3. The validated gene deletions sensitive to *meu1Δ* were assessed for enrichment to the major hierarchical gene ontology (GO) category Molecular Function using YeastEnrichr with *P*-values corrected for multiple testing using the Benjamini -Hochberg false discovery rate of 0.05.

Table S4. The validated gene deletions sensitive to *meu1Δ* were assessed for enrichment to KEGG pathways using YeastEnrichr with *P*-values corrected for multiple testing using the Benjamini -Hochberg false discovery rate of 0.05.

Table S5. Annotation of the chemical-genetic interactions that confer reduced growth or increased growth of cells treated with MTDIA. The growth of the single gene deletion mutants treated with MTDIA relative to vehicle control was determined at a time point when the untreated *xxxΔ* cells were at mid-log. The treated deletion mutants with an average growth <75% (*P*<0.05) or >130% (*P*<0.05) relative to untreated were considered a growth defect or a growth improvement, respectively. Function/process is derived from *Saccharomyces* Genome Database.

Table S6. The validated gene deletions sensitive to MTDIA were assessed for enrichment to the major hierarchical gene ontology (GO) category Biological Process using YeastEnrichr with *P*-values corrected for multiple testing using the Benjamini -Hochberg false discovery rate of 0.05.

Table S7. The validated gene deletions sensitive to MTDIA were assessed for enrichment to the major hierarchical gene ontology (GO) category Molecular Function using YeastEnrichr with *P*-values corrected for multiple testing using the Benjamini -Hochberg false discovery rate of 0.05.

Table S8. The validated gene deletions sensitive to MTDIA were assessed for enrichment to KEGG pathways using YeastEnrichr with *P*-values corrected for multiple testing using the Benjamini -Hochberg false discovery rate of 0.05.

Table S9. Gene deletions that were not viable in MTA as the only sulphur source.

Table S10. Abundance and localization of GFP-tagged proteins in response to MTDIA treatment or MEU1-deficiency. Strains expressing a GFP-tagged protein and dual RFPs were cultured overnight on SD-U+R agar and inoculated into black-walled, clear-bottom 384 well plates to an OD660 of 0.3 in 50 μL volumes of SD-MAU+MTA+RG liquid media with and without MTDIA. Plates were incubated at 30°C for 6 h and the fluorescent signal was detected at 488 nm (GFP) and 561 nm (nuclear localization signal fused to RedStar2 and cytosolic mCherry) using the 60× water immersion lens (NA 1.2) in the high-throughput spinning disk confocal microscope (Evo Tec OPERA, Perkin Elmer). The change in GFP intensity was quantified using Acapella automated image analysis software (Perkin Elmer) and represented as the percent change in GFP abundance relative to control. Localization change was confirmed by visual inspection and validated with independent, reproducible experiments.

Table S11. Degree centrality and betweenness centrality values of genes in the *MTDIA/meu1Δ* network.

Table S12. Strains used in this study.

Table S13. Media used in this study.
