# Supplementary material for: The anti-cancer transition-state inhibitor MTDIA inhibits human MTAP, inducing autophagy in humanized yeast
Source: Dis Model Mech. 2025 Jun 30;18(6):dmm052173. doi: 10.1242/dmm.052173 (PMC12231107; doi:10.1242/dmm.052173)
Supplement: Supplementary information [file dmm-18-052173-s1.pdf]

Table S1. Annotation of the genetic interactions that confer reduced growth or increased growth of meu1Δ. The growth of the double mutants relative to the single haploid parental strains (*meu1Δxxx*Δ*/meu1Δ*)/(*xxxΔ/WT*) × 100 was determined at a time point when the untreated *xxxΔ* cells were at mid-log. Relative to the parental *meu1Δ* and *xxxΔ* single gene deletion strains, the double deletion mutants with an average growth <75% (P<0.05) or >130% (P<0.05) were considered a growth defect or a growth improvement, respectively. Function/process is derived from *Saccharomyces* Genome Database.

Available for download at
https://journals.biologists.com/dmm/article-lookup/doi/10.1242/dmm.052173#supplementary-data

Table S2. The validated gene deletions sensitive to *meu1*Δ were assessed for enrichment to the major hierarchical gene ontology (GO) category Biological Process using YeastEnrichr with *P*-values corrected for multiple testing using the Benjamini -Hochberg false discovery rate of 0.05.

Available for download at
https://journals.biologists.com/dmm/article-lookup/doi/10.1242/dmm.052173#supplementary-data

Table S3. The validated gene deletions sensitive to *meu1*Δ were assessed for enrichment to the major hierarchical gene ontology (GO) category Molecular Function using YeastEnrichr with *P*-values corrected for multiple testing using the Benjamini -Hochberg false discovery rate of 0.05.

Available for download at
https://journals.biologists.com/dmm/article-lookup/doi/10.1242/dmm.052173#supplementary-data

Disease Models & Mechanisms • Supplementary information

Table S4. The validated gene deletions sensitive to *meu1*∆ were assessed for enrichment to KEGG pathways using YeastEnrichr with *P*-values corrected for multiple testing using the Benjamini -Hochberg false discovery rate of 0.05.

Available for download at
https://journals.biologists.com/dmm/article-lookup/doi/10.1242/dmm.052173#supplementary-data

Table S5. Annotation of the chemical-genetic interactions that confer reduced growth or increased growth of cells treated with MTDIA. The growth of the single gene deletion mutants treated with MTDIA relative to vehicle control was determined at a time point when the untreated *xxxΔ* cells were at mid-log. The treated deletion mutants with an average growth <75% ($P<0.05$) or >130% ($P<0.05$) relative to untreated were considered a growth defect or a growth improvement, respectively. Function/process is derived from *Saccharomyces* Genome Database.

Available for download at
https://journals.biologists.com/dmm/article-lookup/doi/10.1242/dmm.052173#supplementary-data

Table S6. The validated gene deletions sensitive to MTDIA were assessed for enrichment to the major hierarchical gene ontology (GO) category Biological Process using YeastEnrichr with *P*-values corrected for multiple testing using the Benjamini -Hochberg false discovery rate of 0.05.

Available for download at
https://journals.biologists.com/dmm/article-lookup/doi/10.1242/dmm.052173#supplementary-data

Table S7. The validated gene deletions sensitive to MTDIA were assessed for enrichment to the major hierarchical gene ontology (GO) category Molecular Function using YeastEnrichr with *P*-values corrected for multiple testing using the Benjamini -Hochberg false discovery rate of 0.05.

Available for download at
https://journals.biologists.com/dmm/article-lookup/doi/10.1242/dmm.052173#supplementary-data

Table S8. The validated gene deletions sensitive to MTDIA were assessed for enrichment to KEGG pathways using YeastEnrichr with *P*-values corrected for multiple testing using the Benjamini -Hochberg false discovery rate of 0.05.

Available for download at
https://journals.biologists.com/dmm/article-lookup/doi/10.1242/dmm.052173#supplementary-data

Table S9. Gene deletions that were not viable in MTA as the only sulphur source.

Available for download at
https://journals.biologists.com/dmm/article-lookup/doi/10.1242/dmm.052173#supplementary-data

Table S10. Abundance and localization of GFP-tagged proteins in response to MTDIA treatment or MEU1-deficiency. Strains expressing a GFP-tagged protein and dual RFPs were cultured overnight on SD-U+R agar and inoculated into black-walled, clear-bottom 384 well plates to an OD660 of 0.3 in 50 µL volumes of SD-MAU+MTA +RG liquid media with and without MTDIA. Plates were incubated at 30°C for 6 h and the fluorescent signal was detected at 488 nm (GFP) and 561 nm (nuclear localization signal fused to RedStar2 and cytosolic mCherry) using the 60× water immersion lens (NA 1.2) in the high-throughput spinning disk confocal microscope (Evo Tec OPERA, Perkin Elmer). The change in GFP intensity was quantified using Acapella automated image analysis software (Perkin Elmer) and represented as the percent change in GFP abundance relative to control. Localization change was confirmed by visual inspection and validated with independent, reproducible experiments.

Available for download at
https://journals.biologists.com/dmm/article-lookup/doi/10.1242/dmm.052173#supplementary-data

Table S11. Degree centrality and betweenness centrality values of genes in the *MTDIA/meu1*Δ network.

Available for download at
https://journals.biologists.com/dmm/article-lookup/doi/10.1242/dmm.052173#supplementary-data

Table S12. Strains used in this study.

Available for download at
https://journals.biologists.com/dmm/article-lookup/doi/10.1242/dmm.052173#supplementary-data

Table S13. Media used in this study.

Available for download at
https://journals.biologists.com/dmm/article-lookup/doi/10.1242/dmm.052173#supplementary-data
